# Biotite: a unifying open source computational biology framework in Python

**DOI:** 10.1186/s12859-018-2367-z

**Published:** 2018-10-01

**Authors:** Patrick Kunzmann, Kay Hamacher

**Affiliations:** Department of Computational Biology and Simulation, TU Darmstadt, Schnittspahnstraße 2, Darmstadt, 64287 Germany

**Keywords:** Open source, Python, NumPy, Structural biology, Sequence analysis

## Abstract

**Background:**

As molecular biology is creating an increasing amount of sequence and structure data, the multitude of software to analyze this data is also rising. Most of the programs are made for a specific task, hence the user often needs to combine multiple programs in order to reach a goal. This can make the data processing unhandy, inflexible and even inefficient due to an overhead of read/write operations. Therefore, it is crucial to have a comprehensive, accessible and efficient computational biology framework in a scripting language to overcome these limitations.

**Results:**

We have developed the Python package Biotite: a general computational biology framework, that represents sequence and structure data based on NumPy*ndarrays*. Furthermore the package contains seamless interfaces to biological databases and external software. The source code is freely accessible at https://github.com/biotite-dev/biotite.

**Conclusions:**

Biotite is unifying in two ways: At first it bundles popular tasks in sequence analysis and structural bioinformatics in a consistently structured package. Secondly it adresses two groups of users: novice programmers get an easy access to Biotite due to its simplicity and the comprehensive documentation. On the other hand, advanced users can profit from its high performance and extensibility. They can implement their algorithms upon Biotite, so they can skip writing code for general functionality (like file parsers) and can focus on what their software makes unique.

**Electronic supplementary material:**

The online version of this article (10.1186/s12859-018-2367-z) contains supplementary material, which is available to authorized users.

## Background

Biology becomes more and more data-driven, with an increasing amount of available genomic sequences and biomolecular structures. In order to make use of this data, a multitude of software has been developed in recent years. Most of these programs have a very specific purpose, like sequence alignment or secondary structure annotation to protein structures. Usually these programs are used via the command line; they are taking some input parameters and files and put their results in output files. It is the task of the user to convert their data of interest into the software specific input format and parse the produced output. This output can be the final result or serve as input for the next program. Depending on the complexity of the user’s initial question this process can be too inflexible and too unhandy to be viable. Furthermore, reading, writing and converting files can yield a significant overhead, increasing the computation time.

These problems can be solved by shifting the workflow from this file-based approach into a scripting language. Here the data needs to be loaded only once and the subsequent analysis is performed based on the framework’s internal representation of the data, with the full flexibility of a programming language. One programming language, suited for this, is Python: It has a simple and easy-to-learn syntax, it is heavily supported by the open source community and the possibility to interface native C code made it to one of the most popular languages for scientific programming.

### Related Work

There are some computational biology frameworks in Python that are already available: MDTraj [1] and MDAnalysis [2] are tools for analysis of trajectories from molecular dynamics simulations. PyCogent [3] and scikit-bio support the analysis of (genomic) sequence data. A framework for working with sequence and structure data combined is Biopython [4], however, this Python package mostly works as *glue* between different programs. The algorithms directly implemented in the Biopython package are limited in scope and efficiency.

We set out to develop a comprehensive computational molecular biology framework for analysis of sequence and structure data, where most of the data can be handled internally, without the usage of additional software. Hence we introduce Biotite, an open source Python package, that can handle the complete bioinformatics workflow, from fetching, reading and writing relevant files to the efficient and intuitive analysis and manipulation of their data.

## Implementation

Biotite is divided into four subpackages: *sequence* and *structure* provide tools for handling sequences or biomolecular structures, respectively. *database* is used for fetching files from biological databases and *application* offers interfaces for external software.

Since computational efficiency is one central aim of the Biotite project, the package makes heavy use of NumPy [5], in places where vectorization is applicable. In cases, where this is not possible, the source code is usually written in Cython [6], resulting in performance comparable to native C code.

### The *sequence* subpackage

Sequences are important objects in bioinformatics. Beside the classical ones, nucleotide and protein sequences, there are for example sequences describing protein structures [7–9] or pharmacophores [10].

In order to account for these special types of sequences, Biotite has a very broad understanding of a sequence: The symbols in a sequence are not limited to single characters (e.g. ’A’,’C’,’G’ and ’T’), but every immutable and hashable Python object can be a symbol, as long as it is present in the alphabet of a sequence. An alphabet represents the set of allowed symbols in the sequence.

**Fig. 1:**
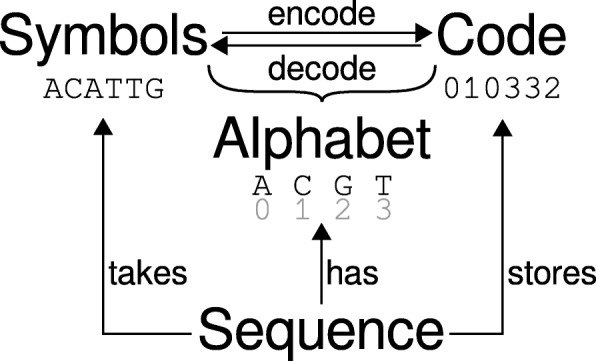
Biotite’s internal representation of sequences. A Sequence object takes symbols as input parameter. Each symbol is encoded into its symbol code, using a Sequence class specific alphabet. The resulting code is then stored as NumPyndarray in the Sequence object

In practice, a sequence is represented by a Sequence instance. When creating a Sequence, each symbol is encoded into an unsigned integer value (*symbol code*) using the Alphabet instance of the Sequence (Fig. 1). The symbol code *c* of a symbol *s* is the index of *s* in the symbol list of the Alphabet instance. Eventually, the symbol codes are stored in a NumPyndarray of the Sequence object. The number of bytes per symbol code in the ndarray is adapted to the number of different symbols in the alphabet. Hence, it is possible to use alphabets with *more* than 256 different symbols typical for byte-oriented mappings traditionally employed.

This approach has multiple advantages:

- Larger variety of possible symbols (multi-character strings, numbers, tuples, etc.)
- Most operations (searches, alignments, etc.) rely on symbol codes and consequently are independent of the actual type of sequence
- Vectorized operations yield a performance boost
- Symbol codes are direct indices for substitution matrices in alignments (discussed below)

#### Nucleotide and protein sequences

**Fig. 2:**
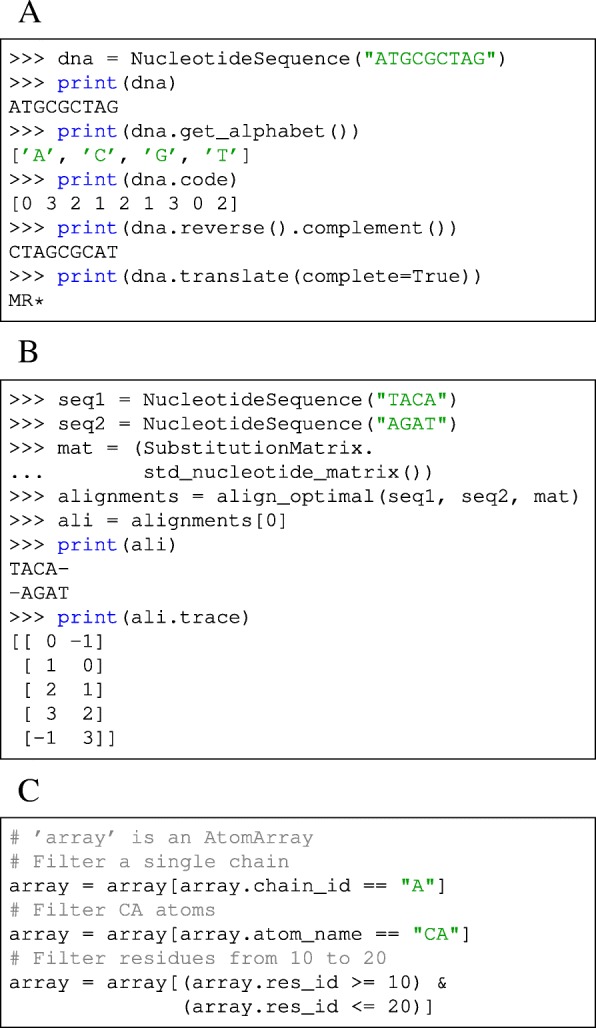
Code examples for Biotite usage. Note that the examples are shortened: Import statements and the AtomArray instantiation are missing. **a** Creation and properties of a NucleotideSequence and its translation into a ProteinSequence. **b** Global alignment of two NucleotideSequence instances. **c** Filtering an AtomArray with *boolean masks*

NucleotideSequence and ProteinSequence are specialized Sequence subclasses that offer common operations for nucleotide and protein sequences (Fig. 2a).

Biotite provides read and write capabilities for the FASTA format, hence FASTA files can be used to load and save nucleotide and protein sequences.

#### Alignments

Biotite offers a function for global [11] and local [12] pairwise sequence alignments with both, linear and affine gap penalties [13] using dynamic programming. Biotite does not use the more complex *divide and conquer* principle [14], hence both, computation time and memory space scale linearly with the lengths of the two aligned sequences. In order to align two Sequence objects a SubstitutionMatrix instance is required. These objects consist of two Alphabet instances, that must fit the alphabets of the aligned sequences, and a score matrix, implemented as 2-dimensional ndarray. The similarity score of two symbols with symbol code *m* and *n*, respectively, is the value of the score matrix at position [*m*,*n*]. This simple indexing operation renders the retrieval of similarity scores highly efficient. In order to decrease the computation time of alignments even more, the underlying dynamic programming algorithm is implemented in Cython.

For a custom SubstitutionMatrix both alphabets can be freely chosen. This implies at first that alignments are independent of the sequence type and secondly that even unequal types of sequences can be aligned. One possible application for alignments of different sequence types is testing the compatibility of a protein sequence to a given protein structure [7]. In addition to custom SubstitutionMatrix instances, all standard NCBI substitution matrices (BLOSUM, PAM, etc.) and the corrected BLOSUM matrices [15] can be loaded.

Alignments in Biotite return Alignment instances. These objects store the *trace* of the aligned sequences, i.e. the indices of the aligned symbols in the original Sequence objects (-1 for gaps) (Fig. 2b).

#### Sequence features

Sequence features describe functional parts of a sequence, for example promoters or coding regions. They consist of a feature key (e.g. *regulatory* or *CDS*), one or multiple locations on the reference sequence and qualifiers that describe the feature in detail. A popular format to store sequence features is the text based GenBank format. Biotite provides a GenBank file parser for conversion of the feature table into Python objects.

#### Visualizations

**Fig. 3:**
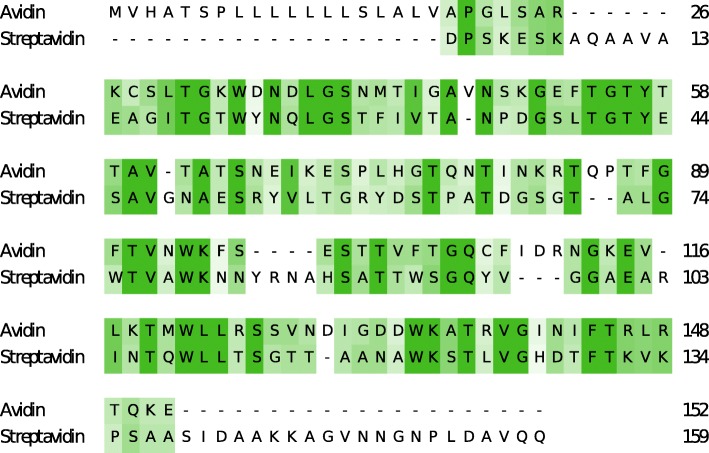
Example sequence alignment visualization. The alignment of an avidin sequence (Accession: CAC34569) with a streptavidin sequence (Accession: ACL82594) is visualized using the AlignmentSimilarityVisualizer

Biotite is able to produce sequence-related visualizations based on matplotlib [16] figures. Hence the visualization can use the various matplotlib backends: It can be displayed on screen, saved to files in different raster and vector graphics formats or embedded in other applications. The base class for all visualizations is the Visualizer class. Its subclasses provide visualization functionality for alignments, sequence logos and sequence annotations. An example alignment visualization, created with the AlignmentSimilarityVisualizer class, is shown in Fig. 3. Further visualization examples are available in the example gallery of the Biotite documentation (Additional file 1).

### The *structure* subpackage

The most basic unit of the representation of a biomolecular structure is the Atom class. An Atom instance contains information about the atom coordinates with a length three ndarray and information about its annotations (like chain ID, residue ID, atom name, etc.). An entire structure, consisting of multiple atoms, is represented by an AtomArray. Rather than storing Atom objects in a list, a much more efficient approach was used: Each annotation category is stored as a length *n*ndarray (*annotation array*) and the coordinates are stored as (*n* ×3) ndarray for a structure with *n* atoms. In some cases the atoms in a structure have multiple coordinates, representing different locations, for example in NMR elucidated structures or in trajectories from molecular dynamics simulations. AtomArrayStack instances represent such multi-model structures. In contrast to an AtomArray, an AtomArrayStack has a (*m* ×*n* ×3) coordinate ndarray for a structure with *n* atoms and *m* models.

Only in a few cases the user will work with single Atom objects. Usually AtomArray and AtomArrayStack instances are used, which enable vectorized (and hence computationally efficient) operations. The atom coordinates and annotation arrays can be simply accessed by calling the corresponding attribute. Furthermore, these objects behave similar to NumPyndarray objects in respect of indexing: An AtomArray or AtomArrayStack can be indexed like an one or two-dimensional ndarray, respectively, with integers, slices, index arrays or *boolean masks*. Thus, annotation arrays in conjunction with *boolean masks* provide a convenient way of filtering a structure, in contrast to the text based selections used in MDAnalysis and MDTraj (Fig. 2c).

The *structure* subpackage can be used to measure distances, angles and dihedral angles, between single atoms, atom arrays, atom array stacks or a combination of them. The broadcasting rules of NumPy apply here.

#### Implemented algorithms

Beside geometric measurements, Biotite offers more complex algorithms for structure analysis: atom-wise accessible surface area calculation (based on the Shrake-Rupley algorithm [17]), structure superimposition (based on the Kabsch algorithm [18]) and secondary structure assignment (based on the P-SEA algorithm [19]) are available. Furthermore, the root-mean-square deviation (RMSD) and fluctuation (RMSF) can be calculated. Currently, the analysis tools focus on protein structures, but specialized functions for structure analysis of nucleic acids are planned for future versions.

#### Reading and writing structure files

AtomArray and AtomArrayStack instances can be loaded from and saved to multiple different file formats. The most basic one is the PDB format, from which only the *ATOM* and *HETATM* records are parsed. An alternative is the modern PDBx/mmCIF format that provides additional information on a structure. Using Biotite, each category in a PDBx/mmCIF file can be converted into a Python dictionary object.

Biotite is also capable of parsing files in the recently published binary MMTF format [20]. This format features a small file size and short parsing times. Instead of relying on the MMTF parser provided by the RCSB (package mmtf-python), Biotite implements an efficient MMTF decoder and encoder written in Cython. Additionally, the conversion from MMTF’s hierarchical data model (chain, residue, atom) into a BiotiteAtomArray or AtomArrayStack is also C-accelerated.

If MDTraj is installed, Biotite is also able to load GROMACS [21] trajectory files (*trr*, *xtc*, *tng*).

### The *database* subpackage

This subpackage is used to download files from the RCSB PDB and NCBI Entrez web server via HTTP requests. Furthermore the RCSB PDB SEARCH service is supported.

### The *application* subpackage

In this subpackage Biotite offers interfaces to external software, including NCBI BLAST [22], MUSCLE [23], MAFFT [24], Clustal-Omega [25] and DSSP [26]. These interfaces wrap the execution of the respective program on the local machine, or use the HTTP-based API (application programming interface) in case of NCBI BLAST. The execution is seamless: Biotite objects, like Sequence or AtomArray are taken as input, and the output (e.g. an alignment) is returned. Writing/reading input/output files is handled internally.

**Fig. 4:**
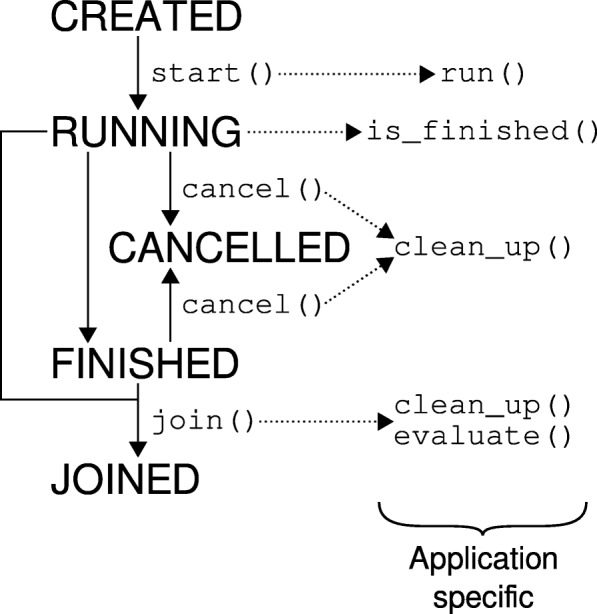
Life cycle of an application. After creation, the Application object is in *CREATED* state. When the user calls start(), the Application enters the *RUNNING* state. When the execution finishes, the state changes to *FINISHED*. The results of the execution are made accessible by calling join(), changing the state to *JOINED*. If the Application is still in the *RUNNING* state then, it is constantly checked whether the execution is finished. The execution can be cancelled using the cancel() method, then the Application ends up in the *CANCELLED* state. This life cycle is equal in all Application subclasses, but each subclass has its own implementation of the application specific methods, that are called on state transition

Application interfaces inherit from the Application superclass. Each Application has a life cycle, based on application states (Fig. 4). After creation, the execution of the Application is started using the start() method. After calling the join() method the results are accessible. If the execution has not finished by then, the Python code will wait until the execution has completed. This approach mimics the behavior of an additional thread: Between the start() and the join() statement other operations can be performed, while the application executes in parallel.

### Software engineering considerations

The Biotite project aims to follow guidelines of good programming practice. The package’s API is fully documented in order to maximize usability. Furthermore, the documentation provides a tutorial and an example gallery. The source code is unit tested with 72% code coverage (calculated via pytest-cov package). However, the actual coverage is greater since Cython files are not considered in the calculation. To ensure that all supported platforms and Python versions are properly supplied with upcoming releases, the project uses *AppVeyor* and *Travis CI* as continuous integration platforms.

## Results and discussion

### Performance of implemented analysis algorithms

**Fig. 5:**
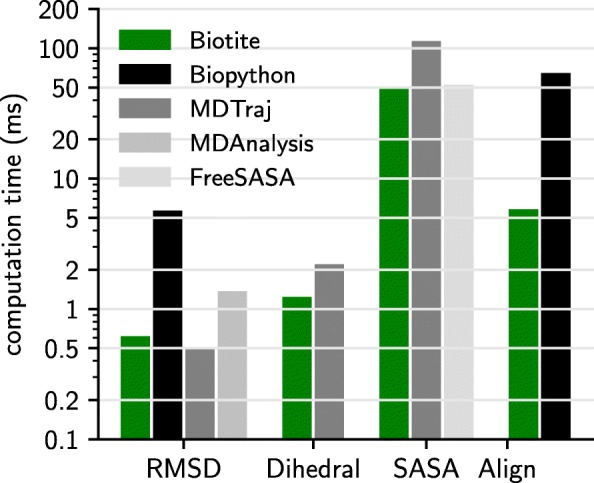
Performance comparison for analysis algorithms. The computation time of performing popular tasks on biological data, starting from the package’s respective internal sequence or structure representation. Note the logarithmic scale. The performance between Biotite, Biopython, MDAnalysis, MDTraj and FreeSASA is shown. A missing bar indicates that the operation is not supported in the respective package. The average of 100 executions was taken. **RMSD**: Superimposition of a structure onto itself and subsequent RMSD calculation (PDB: 1AKI). **Dihedral**: Calculation of the backbone dihedral angles (*ϕ*, *ψ*, *ω*) of a protein (PDB: 1AKI). **SASA**: Calculation of the SASA of a protein (PDB: 1AKI). **Align**: Optimal global alignment of two 1,000 residues long polyalanine sequences

In order to evaluate the capability of Biotite for large scale analyses, the performance of popular tasks was compared to Biopython, MDAnalysis and MDTraj (Fig. 5) (benchmark script in Additional file 2). For structure related tasks the crystal structure of lysozyme was chosen (PDB: 1AKI [27], 1001 atoms), for sequence alignment two 1,000 residues long polyalanine sequences were used. All benchmarks were started from the internal representation of a structure (AtomArray in Biotite) or sequence (Sequence in Biotite), respectively.

One usual task in structural bioinformatics is the superimposition of a structure onto another one (Kabsch algorithm [18]) and the subsequent calculation of the RMSD. In this test case the structure of lysozyme was superimposed onto itself. Biotite, MDTraj and MDAnalysis showed comparable computation time. Compared to that, Biopython was an order of magnitude slower due to to the underlying data representation for structures in Biopython, based on pure Python objects. In consequence the data needs to be time-costly converted into a C-compatible data structure, prior to the actual structure superimposition. This circumstance generally hampers the efficiency when analyzing structures in Biopython: The analysis either requires an expensive conversion or is implemented in pure Python. In the other mentioned packages, including Biotite, the function can be directly executed on the internal ndarray objects. Although this case demonstrates the RMSD computation for a protein structure, Biotite can perform this task also for structures of nucleic acids or any other molecule since the superimposition and RMSD calculation does only depend on atom coordinates.

Another test case was the dihedral angle measurement (*ϕ*, *ψ*, *ω*) of the peptide backbone atoms in the lysozyme structure. Biotite requires approximately half the computation time compared to MDTraj.

The calculation of the solvent accessible surface area (SASA) is relatively time consuming. Both, Biotite and MDTraj, use an implementation of the Shrake-Rupley algorithm [17]. For this benchmark the SASA of the lysozyme structure was calculated, with 1000 sphere points per atom. The measurement shows that Biotite is approximately two times faster than MDTraj. Another benefit of the implementation in Biotite is the ability to use atom radii suited for structures with missing hydrogen atoms [28] like most X-ray elucidated structures. Additionally, the result is compared to the Lee-Richards method [29] implemented in the C-accelerated package FreeSASA. This algorithm uses sphere slices instead of sphere points. The amount of sphere slices was chosen so that the accuracy is equal to the Shrake-Rupley test cases (Additional file 3 and 4). The computation speed is comparable to Biotite.

In regard to sequence data, a frequent operation is the optimal global alignment of two sequences using dynamic programming [11]. Both, Biotite and Biopython, use a C-acclerated function to solve this problem. However, Biotite is an order of magnitude faster in performing this task. The main reason for this is the traceback step, that is C-accelerated in Biotite in contrast to Biopython. Moreover, Biotite uses a substitution matrix to score the alignment, while Biopython only distinguishes between match and mismatch. Although Biopython also supports substitution matrices in alignments, these are based on Python dictionaries. This comes with two disadvantages regarding the computational performance: At first the slow Python API is invoked for every cell in the alignment matrix. Secondly, as Biopython works directly with symbols, the dictionary access with a tuple of symbols is relatively time-consuming compared to the fast indexing operation with symbol codes in Biotite.

Currently, Biotite can only produce pairwise alignments using the *Needleman-Wunsch* [11] and *Smith-Waterman* [12] algorithm, respectively. Although these techniques produce optimal alignments, the computation can be unfeasible for large sequences like entire genomes, as computation time and memory consumption scales linearly with the length of both sequences. Hence, more sophisticated heuristic pairwise alignment methods will be added to the package in future releases. Currently, *Biotite* can perform fast heuristic pairwise alignments using its *NCBI BLAST* [22] interface in the application subpackage.

### Performance of structure file input and output

**Fig. 6:**
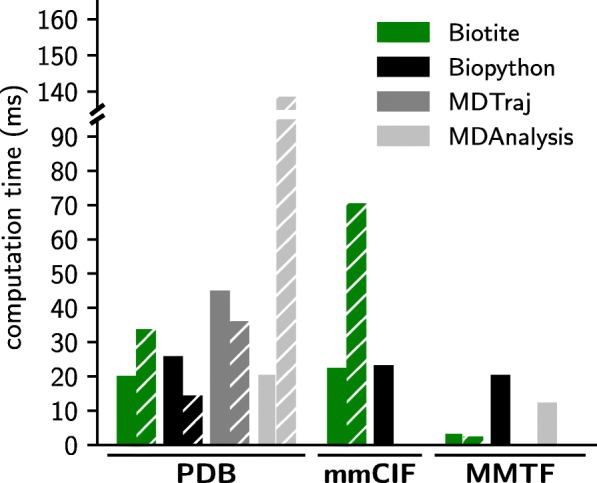
Performance comparison for reading and writing structure files. The computation time of loading a file into the package’s respective internal structure representation (filled bar) and vice versa (hatched bar) is shown. The performance compared between Biotite, Biopython, MDAnalysis and MDTraj is shown. A missing bar indicates that the operation is not supported in the respective package. The structure of an avidin-biotin complex (PDB: 2AVI) was used for computation time measurement. The average of 100 executions was taken

Additionally, the computation time for reading and writing structure files in different formats was compared between Biotite, Biopython, MDAnalysis and MDTraj. The measured time is the time of loading a structure file (PDB: 2AVI [30], 1952 atoms) into the internal representation of the package or saving this representation in a file, respectively. The results are shown in Fig. 6 (benchmark script in Additional file 5).

PDB files are handled in comparable time by Biotite and Biopython: While Biotite is faster in reading PDB files, Biopython has an advantage in writing PDB files. MDAnalysis is very slow in respect of output, MDTraj is slow in PDB file input.

The modern PDBx/mmCIF format is only supported by Biopython and Biotite, while Biopython only supports file parsing, whose performance is comparable to Biotite.

The binary MMTF format shows an exceptional performance in combination with Biotite, with a loading time of 3.7 ms and a saving time of 2.6 ms. The parsing speed is multitudes higher than in Biopython and MDTraj. There are probably two reasons for this. Biotite provides its own MMTF decoder/encoder, which has a higher performance than the official one by the RCSB, since the complete decoding/encoding process is either vectorized or runs in native C code. Furthermore, the conversion of the decoded arrays from the MMTF file into an AtomArray (or AtomArrayStack) and vice versa is accelerated via Cython code. Therefore, MMTF is the preferable format when the user wants to analyze a large amount of structure files with Biotite. Notably, to our knowledge Biotite is the only Python framework that is able to save a structure as MMTF file.

### Benchmark details

The presented benchmarks were run on an *Intel®; Core™ i7-4702MQ* CPU with 8 ×2.20 GHz. The operating system was *Xubuntu* 16.04 and the *CPython* version 3.6.3. The used packages had the following versions:

- biotite 0.7.0
- Cython 0.26.1
- numpy 1.13.3
- matplotlib 2.1.2
- msgpack 0.5.6
- requests 2.18.4
- biopython 1.70
- MDAnalysis 0.17.0
- mdtraj 1.9.1
- freesasa 2.0.3

The average of 100 executions was taken for each benchmark.

## Conclusion

Due to the comprehensive content of Biotite, a large part of the computational molecular biology workflow can be performed with this package: Data of interest can be downloaded from biological databases and subsequently loaded into the Python environment. After analysis or manipulation of the sequence or structure data, it can be saved in various file formats or displayed using the included visualization capabilities. In cases where the required functionality is not directly integrated in the package, Biotite provides means to interface external software in a seamless manner.

To our knowledge, the only computational molecular biology framework in Python that is able to fulfill this function to a similar extent, is Biopython. However, due to the high age of Biopython, the package does not meet established standards of scientific programming in Python, especially the usage of NumPy. Therefore, Biotite can be seen as an efficient alternative.

We think that Biotite is suitable for use by novice programmers, since the extensive tutorial and the code examples give a good introduction into the package. Furthermore, the NumPy-like syntax provides an intuitive way to work with biological data.

Additionally, advanced users benefit from the good performance, that follows from the vectorization via NumPy and the C-acceleration. The fact, that the internal ndarray instances can be directly accessed by the user, makes the Biotite package extensible. Custom algorithms can be easily implemented based on the internal representations of sequence and structure data. If a developer decides to build software upon Biotite, he/she is able to utilize the already implemented file parsers and analysis tools. Hence the development can focus on the unique features of the software.

Biotite is continuously developed. Analysis tools for nucleic acid structures, heuristic sequence alignment methods and interfaces for more biological databases are planned to be added in future versions. Feature requests, bug reports, questions and development in general are handled at https://github.com/biotite-dev/biotite.

## Availability and requirements

**Project name:** Biotite

**Project home page:** https://www.biotite-python.org/

**Operating system(s):** Windows, OS X, Linux

**Programming language:** Python

**Other requirements:** At least Python 3.4, the packages *numpy*, *requests* and *msgpack* must be installed

**License:** BSD 3-Clause

**Any restrictions to use by non-academics:** None

## Additional files

Additional file 1 Biotite documentation. This archive contains the HTML documentation of Biotite 0.7.0. The default entry point is index.html. (7Z 3453 KB)

Additional file 2 Analysis performance benchmark. This Python script contains the benchmark for evaluation of the performance of implemented analysis algorithms. (PY 6 KB)

Additional file 3 Comparison of SASA accuracy. This figure compares the accuracy of the SASA calculation depending on the computation time for the Shrake-Rupley algorithm implementation in Biotite and the Lee-Richards algorithm implementation in FreeSASA. (PDF 200 KB)

Additional file 4 Comparison of SASA accuracy - script. This is the Python script corresponding to Additional file 3. (PY 4 KB)

Additional file 5 Read/write performance benchmark. This Python script contains the benchmark for evaluation of the structure file read/write performance. (PY 8 KB)

Additional file 6 Biotite repository snapshot. This archive contains a snapshot of the Biotite repository at version 0.7.0. (7Z 6668 KB)

## Abbreviations

API: Application programming interface
CDS: Coding DNA sequence
NMR: Nuclear magnetic resonance
RMSD: Root-mean-square deviation
RMSF: Root-mean-square fluctuation
SASA: Solvent accessible surface area
