# Supplementary material for: Biotite: a unifying open source computational biology framework in Python
Source: BMC Bioinformatics. 2018 Oct 1;19:346. doi: 10.1186/s12859-018-2367-z (PMC6167853; doi:10.1186/s12859-018-2367-z)
Supplement: Supplementary file 3 — Comparison of SASA accuracy. This figure compares the accuracy of the SASA calculation depending on the computation time for the Shrake-Rupley algorithm implementation in Biotite and the Lee-Richards algorithm implementation in FreeSASA. (PDF 200 KB) [file 12859_2018_2367_MOESM3_ESM.pdf]

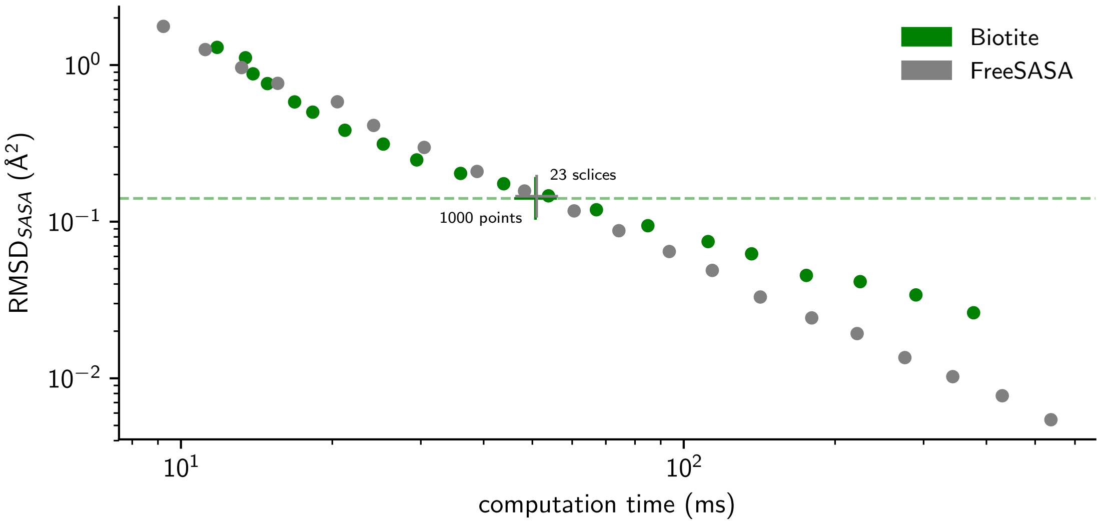

In order to to have a fair comparison of the Shrake-Rupley algorithm implemented in `Biotite` to the Lee-Richards algorithm implemented in `FreeSASA`, the amount of sphere slices for the calculation using `FreeSASA` was chosen, so that the accuracy of the calculation is almost equal to the accuracy of the `Biotite` implementation with 1000 sphere points (default parameter). As measure for the accuracy the root mean square deviation of SASA ($RMSD_{SASA}$) was used, which is defined as

$$RMSD_{SASA} = \sqrt{\frac{1}{N}\sum_{i=1}^{N}(SASA_i - SASA_{ref,i})^2} \tag{1}$$

with N being the amount of atoms, $SASA$ the calculated SASA for the particular amount of points/slices and $SASA_{ref}$ a high precision SASA value used as *correct* SASA. As the calculated SASA converges to the actual SASA with increasing number of points/slices, $SASA_{ref}$ was calculated by employing a number an order of magnitude higher than the tested ones, namely 5000 slices and 100000 points respectively.

For both methods, the SASA was calculated for different numbers of slices/points. Between $10^{0.5}$ and $10^{2.5}$ slices and between $10^{1.7}$ and 10^4 points were tested. The $RMSD_{SASA}$ was calculated and the computation time was recorded (average of 10 runs) for each number.

The $RMSD_{SASA}$ is shown in dependency of the computation time. With 23 slices per sphere `FreeSASA` is almost as accurate as `Biotite` with 1000 sphere points, as indicated by the '+' marker. Hence 23 slices were employed in the benchmark of analysis algorithms. Furthermore, at this level of accuracy both methods do not differ significantly in terms of computation speed. When a higher accuracy is required, the Lee-Richards method of `FreeSASA` performs better.
